# Immunoglobulin G4 Sclerosing Cholangitis: An Unusual Cause of Obstructive Jaundice—Case Report and Literature Review

**DOI:** 10.1155/2018/9602373

**Published:** 2018-08-23

**Authors:** Pragya Shrestha, Brian Le, Brent Wagner, William Pompella, Paras Karmacharya

**Affiliations:** ^1^Department of Medicine, Reading Hospital-Tower Health System, West Reading, PA 19611, USA; ^2^Department of Pathology, Reading Hospital-Tower Health System, West Reading, PA 19611, USA; ^3^Department of Radiology, Reading Hospital-Tower Health System, West Reading, PA 19611, USA; ^4^Department of Rheumatology, Mayo Clinic, Rochester, MN 55905, USA

## Abstract

IgG4-related sclerosing cholangitis (IgG4-SC) is one of the most common extra-pancreatic manifestation of IgG4-related disease (IgG4-RD) and is clinically distinct from primary sclerosing cholangitis (PSC). IgG4-RD is an increasingly recognized immune-mediated fibroinflammatory systemic disease, mostly affecting middle-aged and older male populations that can affect multiple organs. The presence of extra-biliary clinical manifestations of IgG4-RD, such as parotid and lacrimal swelling, lymphadenopathy, autoimmune pancreatitis, and retroperitoneal fibrosis, if present could provide important clues to diagnosis. High serum IgG4 levels, characteristic radiological (e.g., sausage-shaped pancreas or periaortitis) or biopsy findings (high percentage of IgG4+ plasma cells, lymphoplasmacytic infiltrate, storiform fibrosis, or obliterative phlebitis) in the setting of these features is diagnostic of this disease process. However, isolated IgG4-SC might be a diagnostic challenge, and the distinction is important as management of this disorder is vastly different from other causes of cholangitis such as PSC. Systemic corticosteroid therapy is the mainstay of therapy.

## 1. Introduction

Immunoglobulin G4-related sclerosing cholangitis (IgG4-SC) is a relatively uncommon but increasingly recognized entity. It was first described in 2001 by Hamano et al. [1] with a landmark study demonstrating elevated levels of serum IgG4 in patients with sclerosing cholangitis [1–4]. It is characterized by systemic inflammatory and sclerosing lesions with massive infiltration of IgG4-positive lymphocytes involving multiple organ systems, such as the eyes, salivary glands, lacrimal glands, lungs, pancreas, kidneys, retroperitoneum, and vascular system [2, 5]. Sclerosing cholangitis (SC) is one of the common organ manifestations of IgG4-related disease (IgG4-RD), affecting approximately 60% of patients with this systemic disease [5]. It may occur as a part of the systemic manifestation of IgG4-RD, often associated with type 1 autoimmune pancreatitis [1, 2, 6]. Isolated IgG4-SC can occur rarely and pose a diagnostic challenge [5, 6].

## 2. Case Presentation

An 81-year-old male presented to the clinic with yellowish discoloration of skin and urine for 2 weeks. He denied any fever, abdominal pain, nausea, vomiting, melena, hematochezia, or acholic stools. Past medical history was significant for hypertension, hyperlipidemia, diabetes mellitus type II, coronary artery disease, and chronic kidney disease stage IV. He reported recent loss of appetite but denied any significant weight changes. Ultrasound ordered by primary care physician showed intra- and extra-hepatic biliary dilation with distension of gall bladder without cholelithiasis. He was sent to the emergency department (ED) for further evaluation.

On examination, blood pressure was 133/60 mmHg, heart rate was 75 beats per minute, respiratory rate was 23 breaths per minute, temperature was 97.7°F, and oxygen saturation was 98% in room air. He had mild icteric sclera, and chronic venous stasis changes in bilateral lower extremities were noted. Bowel sounds were normal, and no hepatosplenomegaly or abdominal tenderness was noted on exam.

Laboratory investigations showed a hemoglobin count of 11 g/dl, white blood cell count of (WBC) 3800 cell/mm^3^, and platelet count of 214,000/mm^3^. Alanine aminotransferase (ALT) and aspartate aminotransferase (AST) were elevated at 326 and 321 IU/L, respectively. Total bilirubin was 3.1 mg/dl with direct bilirubin of 1.8 mg/dl. Alkaline phosphatase (ALP) was 1,219 IU/L with lipase 250 IU/L. Renal function tests were at baseline at 1.72 mg/dl (baseline 1.7–1.9 mg/dl). Recent upper and lower endoscopy (1 month earlier) did not show significant abnormalities, except for mild antral gastropathy. A computed tomography (CT) scan of abdomen and pelvis revealed stable pelvic adenopathy with largest lymph node measuring 4.4 × 1.7 cm (which was noted on the earlier CT scan as well). The pancreatic tissue and abdominal vessels appeared normal. With concern for underlying malignancy, a lymph node biopsy was performed. Endoscopic retrograde of cholangiopancreatography revealed distal common bile duct stricture of 3 cm without obstruction, for which a biliary stent was placed. No pancreatic lesions were observed, and biliary brushings were negative for malignancy.

**Figure 1:**
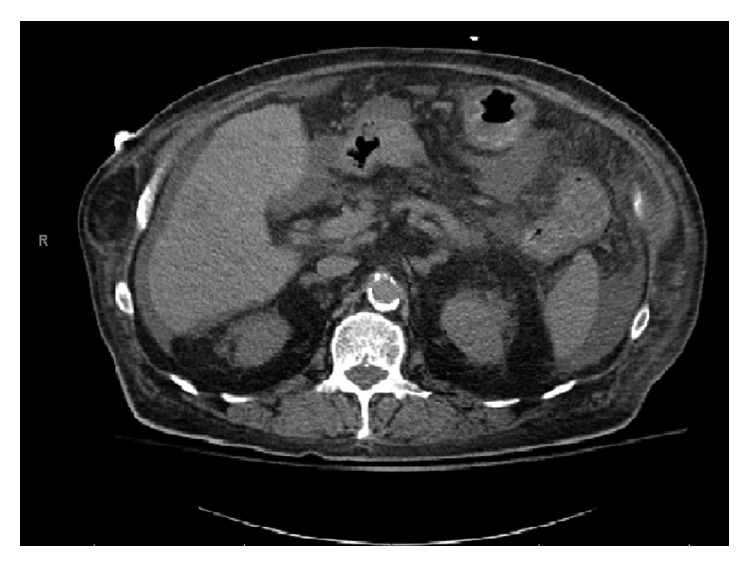
Gall bladder wall thickening with pericholecystic inflammatory stranding concerning for acute cholecystitis with normal pancreas.

The patient returned to the ED two months later; this time with a fever (102°F), nausea, and right upper quadrant pain for 2 days. Complete blood counts revealed elevated white cell count of 18,700/mm^3^. AST, ALT, and bilirubin levels were within normal limits, and ALP was elevated at 127 IU/L. The CT scan of abdomen revealed intrahepatic biliary ductal dilatation and gall bladder wall thickening with pericholecystic inflammatory stranding with normal pancreas (Figure 1). He was started on broad-spectrum antibiotics with piperacillin-tazobactam. Urgent surgical decompression with percutaneous drain placement was performed for stent blockage (with re-stent placement) and bile duct stricture from unknown etiology.

**Figure 2:**
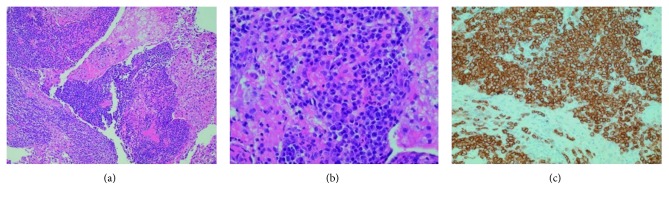
Biopsy fragment of right iliac lymph node showing sinus histiocytic aggregates interlaced with lymphocytes and plasma cells. (a) H&E stain, 100x original magnification. Small cells within the lymph nodes, showing morphologic features of plasma cells, characterized by an oval cellular morphologic, and eccentrically placed nuclei. (b) H&E stain, 200x original magnification. Majority of cellular constituents confirmed to be plasma cells, as demonstrated by diffuse reactivity for CD138 by immunohistochemistry. (c) 200x original magnification.

**Figure 3:**
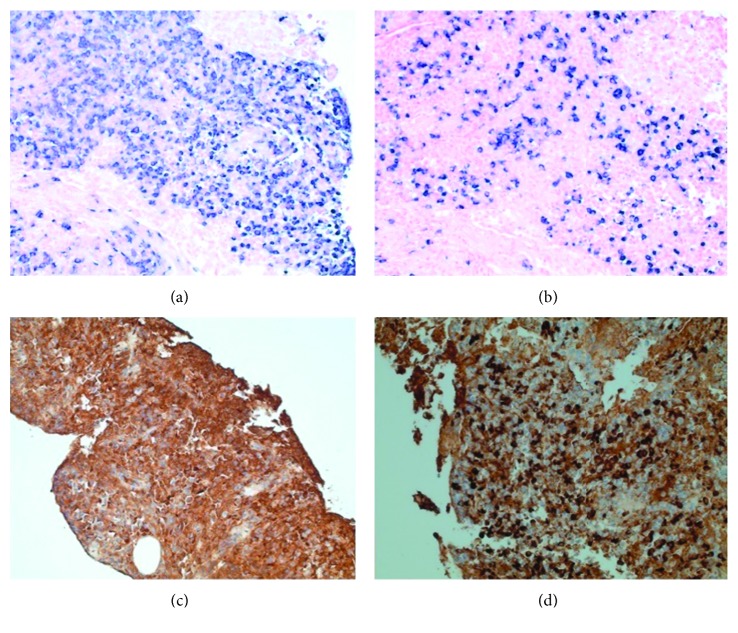
Chromogenic in situ hybridization showing a mixture of kappa-bearing plasma (a) and lambda-bearing plasma (b) cells, with an approximate kappa to lambda ratio of 3–4 to 1, appropriate for a physiologic/reactive phenomenon, without evidence of obvious restriction. Plasma cells expressed predominantly IgG (c); of these, the majority (over 60%) were immunoreactive for IgG4 (d) (200x original magnification).

Meanwhile, histopathology from the earlier right iliac lymph node biopsy revealed sinus histiocytic aggregates interlaced with lymphocytes and plasma cells (Figure 2(a)), majority of cellular constituents being plasma cells, characterized by oval cellular contours and eccentrically located nuclei (Figure 2(b)); this was confirmed by diffuse immunoreactivity for CD138 (Figure 2(c)). Chromogenic in situ hybridization for kappa light chain (Figure 3(a)) and lambda light chain (Figure 3(b)) showed a mixture of kappa and lambda-bearing cells (approximate kappa to lambda ratio of 3 : 1); this finding suggested that the plasma cells were reactive in nature, arguing against the possibility of a plasma cell neoplasm. In situ hybridization for EBV-encoded RNA (EBER) was interpreted as negative.

As there was no evidence to suggest a lymphoma or a plasma cell neoplasm, a diagnosis of IgG-related disease was considered. Additional immunohistochemistry showed that the plasma cells present expressed predominantly IgG (Figure 3(c)); of these, the majority (over 60%) were reactive for IgG4 (Figure 3(d)). Serum protein electrophoresis and immunofixation showed polyclonal increase in IgG with no monoclonal proteins. Serum immunoglobulin (Ig) G levels were high (3390 mg/dl) with normal IgA and IgM. IgG subclasses were also high with IgG1 of 1800, IgG2 975, IgG3 324, and IgG4 729 mg/dl.

**Figure 4:**
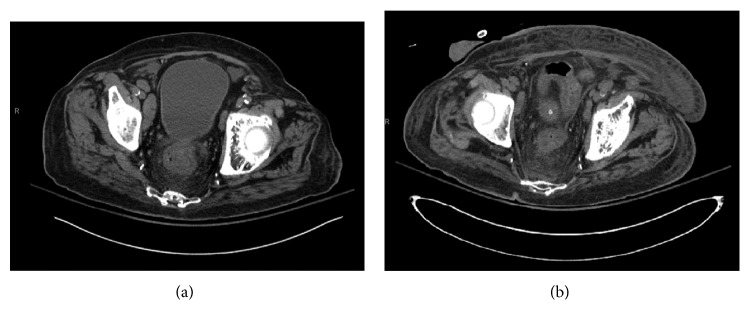
Top normal-sized pelvic lymph nodes decreased in size in repeat CT (4(b)) compared to previous CT (4(a)). The largest right external iliac lymph node measures 1.8 × 0.7 cm.

**Figure 5:**
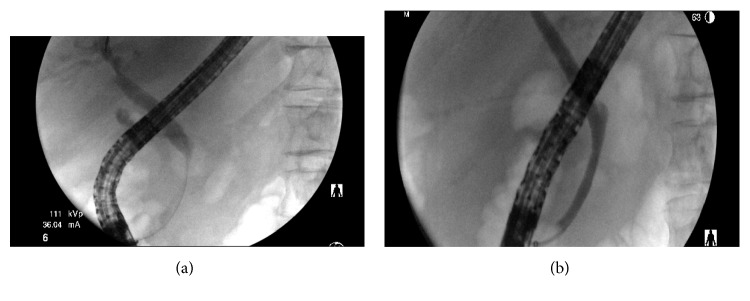
Endoscopic retrograde cholangiography showing presence of stricture in the biliary duct pre-ERC (a) compared to post-ERC (b).

A diagnosis of IgG4-related sclerosing cholangitis was made, and he was started on prednisone 40 mg daily for 6 weeks. A follow-up CT scan of abdomen after a month revealed significant reduction in the size of his pelvic lymph nodes with largest lymph node measuring 1.8 × 0.7 cm (Figure 4). Endoscopic retrograde cholangiography (ERC) performed revealed resolution of stricture (Figure 5). The biliary stent was removed following resolution of the stricture. He remains on prednisone 20 mg daily with close rheumatology follow-up. Rituximab therapy is being considered as a steroid-sparing agent.

## 3. Description

Although reported as a relatively rare disease with a prevalence of 100 per 100,000 adults based on limited studies done in Japan [7], the prevalence of IgG4-RD is likely higher due to underdiagnoses. Fortunately, increasing recognition among various specialties due to its multisystem involvement has been noted in recent years [8]. High index of suspicion for IGg4-RD should be raised in the setting of a constitution of clinical features such as pancreatitis (present in 60% of IgG4-RD) of unknown origin, sclerosing cholangitis (13% of IgG4-RD), or bilateral salivary and/or lacrimal gland enlargement (34% of IgG4-RD) [5, 9]. Proximal IgG4-SC (hilar and intrahepatic bile duct) may occur solely or in association with pancreatitis, whereas isolated lower duct IgG4-SC, as in our case, is rare and might pose a diagnostic challenge. It is more commonly observed with IgG4-RD affecting organs outside the pancreatobiliary system [5].

The differentiation of IgG4-SC from primary sclerosing cholangitis (PSC) and cholangiocarcinoma is clinically very important as IgG4-SC usually has a good response to steroid therapy [10]. IgG-SC patients are generally older than PSC patients, with more than 90% being diagnosed at or above 60 years of age [5, 6, 8, 9]. Also, unlike other autoimmune conditions, IgG4-SC is more common in males (M : F = 4 : 1). Imaging features suggestive of PSC are multifocal intrahepatic duct involvement with beaded pattern secondary to short segmental strictures, whereas IgG4-SC has prominent wall thickening in the bile duct [10]. Similarly, imaging features of cholangiocarcinoma include solitary lesion with irregular margins with eccentric wall thickening and invisible bile duct lumen in the involved segment [10, 11].

The Japanese IgG4-RD committee has recently published a diagnostic criterion for IgG4-SC (2012) based on a combination of 4 features: (1) characteristic biliary imaging findings—diffuse swelling or organ mass, (2) elevation of serum IgG4 concentrations (≥135 mg/dl), (3) coexistence of IgG4-related diseases, and (4) characteristic histopathological features of steroid therapy are optional criteria to confirm accurate diagnosis of IgG4-SC [8, 12]. Typical histopathologic features of IgG4-RD include marked lymphocytic and plasmacyte infiltration with high percentage of IgG4-positive plasma cells, storiform fibrosis, and obliterative phlebitis [13]. In our case, the first two histopathologic features were observed.

Glucocorticoids remain the mainstay of therapy for remission induction in all patients with active IgG4-RD. Retrospective studies performed have shown good response to immunosuppression (prednisone at a dose of 30–40 mg per day), which generally leads to a rapid induction of disease remission [2, 5]. In refractory cases or patients with difficulty in tapering prednisone (usually below 5 mg/day), addition of steroid-sparing agents such as rituximab might be indicated. Although the natural history and long-term outcomes are not well understood, majority of patients tend to relapse, and most patients have a chronic disease course [14]. Hence, close follow-up with rheumatology is recommended.

## 4. Conclusion

Isolated IgG4-SC is a difficult entity to diagnose with vague presentation and overlapping symptoms with PSC, cholangiocarcinoma, other autoimmune conditions, and malignancy. Appropriate clinical, serologic, radiologic, and pathologic features may aid in diagnosis, although none in isolation is diagnostic. Glucocorticoids are the mainstay of therapy, and rituximab might be added as a steroid-sparing agent, especially in refractory or steroid-dependent cases. Close follow-up is recommended due to its chronic and relapsing course.
